# Genetic Variants in Telomere-Maintenance Genes and Bladder Cancer Risk

**DOI:** 10.1371/journal.pone.0030665

**Published:** 2012-02-17

**Authors:** Joshua Chang, Colin P. Dinney, Maosheng Huang, Xifeng Wu, Jian Gu

**Affiliations:** 1 Department of Epidemiology, The University of Texas MD Anderson Cancer Center, Houston, Texas, United States of America; 2 Department of Urology, The University of Texas MD Anderson Cancer Center, Houston, Texas, United States of America; Tulane University Health Sciences Center, United States of America

## Abstract

Telomeres are critical in maintaining genomic stability. Genetic variants in telomere pathway genes may affect telomere and telomerase function, and subsequently cancer risk. We evaluated 126 SNPs from 10 genes related to telomere regulation in relation to bladder cancer risk. Five SNPs, 4 from TEP1 gene and 1 from PINX1 gene, were found to be highly significant (P<0.01). Out of these, the most significant association was found in rs2228041 of TEP1 (OR 1.66, 95% CI 1.19–2.31) while rs1469557 of PINX1 had a protective effect (OR 0.75, 95% CI 0.61–0.93). Haplotype analysis showed that a TEP1 haplotype consisting of the variant alleles of 7 SNPs exhibited a 2.28 fold increased risk (95% CI 1.13–4.60). We then performed cumulative analysis of multiple risk variants, as well as Classification and Regression Tree (CART) to look for gene-gene interactions. In cumulative effect analysis, the group with 4–5 risk variants had an OR of 2.57 (95% CI = 1.62–4.09) versus the reference group with 0 risk variants. The CART analysis categorized individuals into five subgroups with different bladder cancer risk profiles based on their distinct genotype background. To our knowledge, this is one of the largest, most comprehensive studies on bladder cancer risk concerning telomere-regulating pathway gene SNPs and our results support that genetic variations of telomere maintenance modulate bladder cancer risk individually and jointly.

## Introduction

Telomeres form the ends of chromosomes and consist of nucleotide TTAGGG sequence repeats and the associated protein complex shelterin in mammalian cells [1], [2]. Telomeres prevent the ends of chromosomes from being recognized as double-strand breaks and are vital for genomic integrity, preventing end-to-end fusion, nucleolytic degradation, and atypical recombination [3]. The shelterin complex, composed of six core proteins, helps to prevent recognition of telomeres by DNA damage repair pathways [2], and also modulates telomerase activity [2], [4]. Telomerase, a specialized reverse transcriptase, adds TTAGGG repeats to elongate telomeres using an internal RNA template [5].

In somatic cells, telomeres progressively shorten by 30 to 200 bp after each mitotic division due to incomplete replication of telomeric DNA by DNA polymerases, known as the end-replication problem [6]. When telomere length becomes critically short, loss of telomere protection results in initiation of cell senescence and eventually leads to apoptosis, triggering DNA damage response at telomeric chromosome ends which are recognized as double-strand breaks [7]. However, such a process results in strong selection for cells with defective DNA damage responses that can bypass this telomere checkpoint [8]. Unlimited proliferation is gained through upregulation of telomerase that compensates for telomere erosion in cancer cells [9]. Telomerase activity has been detected in ∼85% of cancers, and is a characteristic of most cancers [10], [11]; in several TERT-transgenic mouse models, constitutive telomerase expression increased cancer incidence [12]. Loss of telomere function and continued proliferation leads to end-to-end fusions, broken chromosomes, breakage-fusion bridge cycles, and general genetic instability; the result is accelerated genetic changes responsible for further growth advantages and cancer cell development [13].

The inverse relationship between telomere length and age has also been well documented [9]. The rate of telomere attrition is dependent on many factors: smoking, obesity, unhealthy lifestyle, and oxidative stress are all associated with shorter telomeres [14]. Genetics strongly influence telomere length and genetic heritability of leukocyte telomere length has been estimated at around 80% [15]. Telomere shortening has been associated with increased risks of several cancers, with bladder cancer being the most consistent [16]. Previous studies have found that single-nucleotide polymorphisms (SNPs) in telomere pathway genes associated with altered cancer risk; for example, a recent study found variants of telomerase-associated protein (*TEP1*) associated with increased bladder cancer risk [17]. In this current study, we took a pathway-based approach to evaluate the association of haplotype tagging and functional SNPs in critical telomere maintenance genes, including shelterin component, telomerase, and telomere/telomerase associated genes, with bladder cancer risk in a large case-control study.

## Results

### Patient characteristics

A total of 803 Caucasian patients diagnosed with bladder cancer and 803 Caucasian control subjects were included in this study (Table 1). Cases and controls were matched on sex (p = 0.95) and age (p = 0.10). Cases had a higher percentage of current smokers (47.45%) versus controls (23.29%, p = 5.15E-21), and among ever smokers, cases had a higher mean pack year (43.02±30.73 years) versus controls (29.92±27.87 years, p = 2.78E-12).

**Table 1:** Distribution of select characteristics among study subjects.

Category	Subcategory	Control subjects	Case patients	P value
**Sex, No. (%)**	Male	639 (79.58)	640 (79.70)	
	Female	164 (20.42)	163 (20.30)	0.9506
**Smoking status, No. (%)**	Never	355 (44.21)	212 (26.40)	
	Former	381 (47.45)	404 (50.31)	
	Current	67 (8.34)	187 (23.29)	5.15×10^−21^
**Age, Mean (SD)**		63.82 (10.88)	64.73 (11.13)	0.0982
**Pack year, Mean (SD)**		29.92 (27.87)	43.02 (30.73)	2.78×10^−12^

### Risk associated with individual SNPs

Among the 126 assayed SNPs, 24 SNPs (19%) were significantly associated with bladder cancer risk at the 5% level. After removing SNPs with high linkage (r^2^>0.8 between a few tagging SNPs and coding SNPs), 18 SNPs remained for the subsequent analysis (Table 2). It is noteworthy that 7 SNPs in both the *TEP1* and *PINX1* gene were significant at p<0.05. All of the SNPs in *TEP1* were associated with increased risk, and all SNPs except one in *PINX1* were associated with reduced risk of bladder cancer. One SNP in POT1, one in TRF2, and two in TNKS were also significant. Since multiple testing was performed, we calculated the Q value (a false discovery rate adjusted P value) to adjust the significance level for individual SNPs and the Q values for these 18 SNPs were between 0.08 and 0.12 (data not shown).

**Table 2:** Individual SNPs associated with bladder cancer risk.

SNP	Gene	Chr	Model	OR (95CI)	P value
**rs1469557**	PINX1	8	DOM	0.75 (0.61–0.93)	**0.0077** *
rs17152584	PINX1	8	DOM	0.76 (0.60–0.96)	0.0186
r s6995541	PINX1	8	ADD	0.82 (0.70–0.97)	0.0199
rs9657541	PINX1	8	DOM	0.78 (0.63–0.97)	0.0220
rs11250080	PINX1	8	REC	0.66 (0.46–0.95)	0.0250
rs7826180	PINX1	8	REC	0.60 (0.38–0.96)	0.0301
rs2409655	PINX1	8	DOM	1.24 (1.01–1.52)	0.0392
rs4360236	POT1	7	DOM	0.75 (0.58–0.97)	0.0261
**rs2228041**	TEP1	14	DOM	1.66 (1.19–2.31)	**0.0023**
**rs2228026**	TEP1	14	DOM	1.72 (1.20–2.44)	**0.0025**
**rs1713418**	TEP1	14	REC	1.42 (1.10–1.83)	**0.0075**
**rs2297615**	TEP1	14	ADD	1.24 (1.05–1.46)	**0.0097**
rs2229101	TEP1	14	DOM	1.46 (1.06–2.00)	0.0192
rs2104978	TEP1	14	DOM	1.40 (1.03–1.91)	0.0309
rs1713440	TEP1	14	ADD	1.17 (1.01–1.35)	0.0403
rs251796	TERF2	16	DOM	1.53 (1.08–2.16)	0.0154
rs7825818	TNKS	8	DOM	1.32 (1.06–1.64)	0.0118
rs10503380	TNKS	8	ADD	1.21 (1.03–1.43)	0.0234

*SNPs with P-values<0.01 were bolded.

Of particular interest, 5 SNPs were found to be highly significant (p<0.01), 4 from *TEP1* and 1 from *PINX1*. The breakdown of these SNPs is found in Table 3. Out of these, the most significant association was found in rs2228026 of *TEP1* (OR 1.72, 95% CI 1.20–2.44), while the rs1469557 of *PINX1* had a protective effect (OR 0.75, 95% CI 0.61–0.93). To explore interactions of genetic variants with smoking status, age, and tumor stage, we performed stratified analysis on these 5 highly significant SNPs, but we did not notice any significant difference of ORs in never and ever-smokers, in old aged and young aged individuals, and in non-muscle invasive and muscle-invasive tumors (data not shown).

**Table 3:** Logistic regression analysis of highly significant SNPs (P<0.01).

SNP	Gene	Genotype	Cases (%)	Controls (%)	OR (95CI)	P value	Q value
zrs2228041	TEP1	GG	734 (91.41)	694 (86.64)	1 (ref)		
		GA	67 (8.34)	107 (13.36)	1.69 (1.21–2.36)		
		AA	2 (0.25)	1 (0.12)	0.52 (0.04–7.38)		
		AA+GA vs. GG			1.66 (1.19–2.31)	0.0023	0.089
rs2228026	TEP1	TT	744 (92.65)	709 (88.51)	1 (ref)		
		TC	57 (7.10)	93 (11.61)	1.75 (1.23–2.50)		
		CC	2 (0.25)	1 (0.12)	0.52 (0.36–7.38)		
		CC+TC vs. TT			1.72 (1.20–2.44)	0.0025	0.089
rs1713418	TEP1	TT	268 (33.37)	244 (30.46)	1 (ref)		
		TC	397 (49.44)	379 (47.32)	1.02 (0.81–1.29)		
		CC	138 (17.19)	180 (22.47)	1.43 (1.07–1.92)		
		CC+TC vs. TT			1.42 (1.10–1.83)	0.0075	0.110
rs2297615	TEP1	TT	475 (59.15)	428 (53.43)	1 (ref)		
		TA	284 (35.37)	314 (39.20)	1.21 (0.98–1.50)		
		AA	44 (5.48)	61 (7.62)	1.61 (1.06–2.46)		
		Additive			1.24 (1.05–1.46)	0.0097	0.110
rs1469557	PINX1	CC	485 (60.40)	532 (66.42)	1 (ref)		
		CT	283 (35.24)	244 (30.46)	0.76 (0.61–0.94)		
		TT	35 (4.36)	27 (3.37)	0.70 (0.41–1.20)		
		TT+CT vs. CC			0.75 (0.61–0.93)	0.0077	0.110

Because many SNPs of the *TEP1* gene were associated with increased risk, and 4 out of 5 highly significant SNPs were from *TEP1*, we performed haplotype analysis on the 7 significant *TEP1* SNPs (Table 4). Compared to the halpotype with the wild-type alleles at all the 7 SNPs, the haplotype containing the variant alleles at all the 7 SNPs exhibited a significantly increased risk (OR 2.28, 95% CI 1.13–4.60, p = 0.022). None of the other haplotypes showed significance in affecting bladder cancer risk.

**Table 4:** Haplotype analysis of *TEP1* gene.

Haplotype group*	Controls (%)	Cases (%)	OR (95CI)	P value
W-W-W-W-W-W-W	551 (46.85)	482 (43.11)	1 (ref)	
W-W-W-W-W-W-M	187 (15.90)	178 (15.92)	1.12 (0.87–1.43)	0.3836
M-W-W-W-W-W-W	136 (11.56)	128 (11.45)	1.06 (0.80–1.40)	0.6750
M-W-W-M-W-W-W	131 (11.14)	123 (11.00)	1.08 (0.80–1.44)	0.5921
M-W-W-W-W-W-M	54 (4.59)	61 (5.46)	1.32 (0.88–1.96)	0.1751
M-W-W-M-W-W-M	49 (4.17)	60 (5.37)	1.42 (0.95–2.14)	0.0896
**M-M-M-M-M-M-M**	13 (1.11)	25 (2.24)	2.23 (1.13–4.60)	**0.0220** **
M-M-M-M-M-M-W	14 (1.19)	17 (1.52)	1.34 (0.63–2.84)	0.4467
M-M-W-M-W-W-M	12 (1.02)	16 (1.43)	1.81 (0.84–3.91)	0.1304
Other	29 (2.47)	28 (2.50)	1.07 (0.62–1.84)	0.8083

*Order of SNPs: rs1713418, rs2104978, rs17211355, rs2297615, rs2228041, rs2228026, rs1713440.

**Haplotype with a P-value<0.05 was bolded.

### Combined effect of multiple SNPs

The 5 highly significant SNPs (p<0.01) were considered for cumulative effects of SNPs on bladder cancer risk. We found a significant gene-dosage effect for increasing bladder cancer risk with increasing number of unfavorable genotypes (p for trend = 3.31E-06), and patients were categorized into 3 risk groups according to number of unfavorable genotypes. Compared to individuals with no unfavorable genotypes, the risk of bladder cancer progressively increased with addition of unfavorable genotypes, with ORs of 1.2 (95% CI 0.92–1.62) for low-risk group with 1 unfavorable genotype, 1.64 (95% CI 1.22–2.21) for medium-risk group with 2–3 unfavorable genotypes, and 2.57 (95% CI 1.62–4.09) for high-risk group with 4–5 unfavorable genotypes (Table 5).

**Table 5:** Cumulative analysis of the top 5 most significant SNPs.

Genotypes	Controls (%)	Cases (%)	OR (95CI)	P value
0	171 (21.30)	129 (16.08)	1 (ref)	
1	364 (45.33)	327 (40.77)	1.2 (0.92–1.62)	0.1670
2∼3	228 (28.39)	272 (33.92)	1.64 (1.22–2.21)	0.0011
4∼5	40 (4.98)	74 (9.22)	2.57 (1.62–4.09)	6.46×10^−5^
p for trend				3.31×10^−6^

### CART Analysis

**Figure 1:**
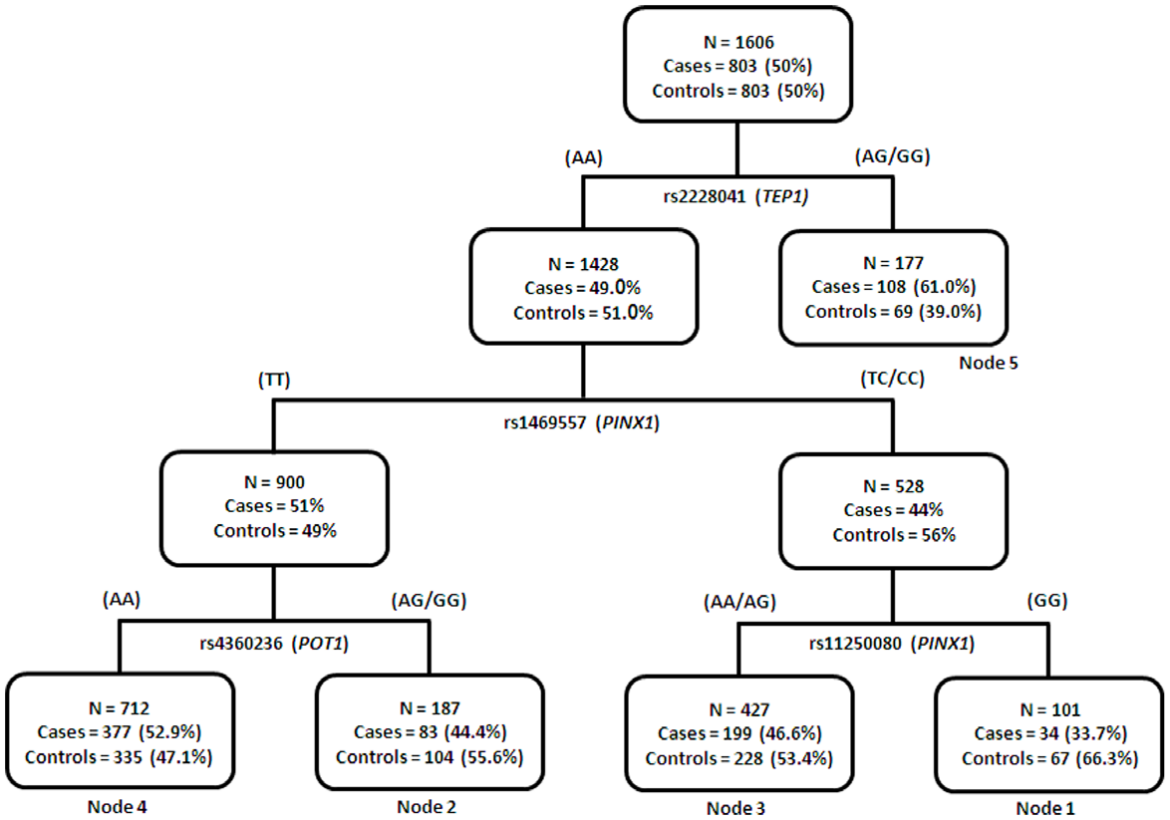
The tree structure of the CART analysis of interaction effects between the 18 top variants (P<0.05, **Table 2**) in modulating bladder cancer risk.

All significantly associated SNPs (Table 2) were analyzed for potential gene-gene interactions through CART analysis. The initial split was at rs2228041 of *TEP1*, the most significant SNP out of those evaluated for bladder cancer risk. The final tree had 5 terminal nodes (Figure 1). Table 6 summarizes the risk estimates for individuals in each terminal node. Node 1 (N = 101), used for reference, had the lowest risk and comprised of patients who were GG for rs11250080 on *PINX1*, TC/CC for rs1469557 on *PINX1*, and AA for rs2228041 on *TEP1*. Compared to individuals in node 1, the other nodes were associated with increased bladder cancer risk with ORs ranging from 1.74 to 3.28 based on distinct genotype combinations. Individuals in node 5 (N = 177) with AG/GG for rs2228041 on *TEP1* had the highest risk (OR 3.28, 95% CI 1.94–5.57).

**Table 6:** Logistic regression analysis of terminal nodes in CART analysis.

CART node	Controls (%)	Cases (%)	OR (95CI)	P value
1	67 (8.34)	34 (4.24)	1 (ref)	
2	104 (12.95)	83 (10.36)	1.74 (1.04–2.93)	0.0363
3	228 (28.39)	199 (24.84)	1.82 (1.14–2.90)	0.0123
4	335 (41.72)	377 (47.07)	2.35 (1.49–3.69)	2.17×10^−4^
5	69 (8.59)	108 (13.48)	3.28 (1.94–5.57)	1.02×10^−5^
p for trend				1.28×10^−6^

## Discussion

This study evaluated the association between a set of SNPs in telomere maintenance genes and bladder cancer risk. Eighteen significant SNPs were found: among SNPs with very significant association (p<0.01), 4 were from telomerase protein component 1 (*TEP1*) and 1 was from PIN2/TRF1-interacting protein 1 (*PINX1*). We also found a significant cumulative effect of multiple SNPs, and potential gene-gene interactions concerning risk.

Telomere shortening and telomerase activation is linked to genomic instability and tumorigenesis. Many studies showed that shorter telomere length is associated with higher risk of several cancers [18], [19], [20], [21], [22], [23], [24], with the strongest evidence in bladder cancer [25]. Telomerase is active in most cancers and is critical for tumorigenesis. It is likely that the studied genetic variants affect cancer risk through changes in mechanisms involving telomere regulation, telomere length, or telomerase function.

Previous studies have shown selected genetic variants in genes of telomere pathway and bladder cancer risk [26], [27]. *TEP1* is a component of the ribonucleoprotein complex and binds to telomerase. A SNP (rs1760897) in *TEP1* has recently been associated with an increased risk of bladder cancer [17]. We also genotyped this SNP in this current study and found this SNP was associated with a borderline significantly increased risk of bladder cancer (OR 1.17, 95% CI 0.94–1.45 and OR 1.27, 95% CI 0.91–1.79 for heterozygous and homozygous variant genotypes, respectively; p for trend = 0.08). In addition, in our study, we found 7 *TEP1* SNPs associated with increased bladder cancer risk. The most significant SNP was rs2228041. This SNP is a non-synonymous SNP, Arg1155Gln. Changing a strong basic amino acid (arginine) to a neutral amino acid (glutamine) is likely to affect protein structure and function. Future studies are needed to determine how this *TEP1* SNP affects TEP1 function, telomerase activity, and bladder cancer risk. Our haplotype analysis also supports the role of TEP1 in bladder cancer etiology.

In addition to *TEP1*, we found high significance in a SNP on the *PINX1* gene and lower bladder cancer risk. *PINX1* regulates telomerase function and can directly bind to TERT and inhibit telomerase activity; inhibition of *PINX1* increases telomerase activity, while overexpression does the opposite [28]. A previous study showed that *PINX1* inhibition leads to aberrant telomerase activation and telomere elongation, compromising telomere function and causing chromosomal instability, and there is evidence supporting the role of *PINX1* as a tumor suppressor, acting through a telomerase-dependent mechanism [29]. Our findings provide further support that *PINX1* is a potential tumor suppressor. Potentially, genetic variation of the *PINX1* gene could alter cancer risk through mechanisms of telomere regulation, and more studies are warranted to evaluate genetic variants within the *PINX1* gene and association with bladder cancer risk, as well as to define how *PINX1* regulates telomeres through telomerase-dependent or independent mechanisms.

We performed cumulative analysis of multiple SNPs. Although the analyzed SNPS individually had moderate effect on bladder cancer risk, we found a stronger cumulative effect. These results confirm the multigenicity of bladder cancer, as noted in previous studies [30], [31], [32], and identification of multiple risk variants could further improve risk prediction. As well, we performed CART analysis to explore high order gene-gene interactions among SNPs. Since bladder cancer is a multi-factor disease, interactions between genetic variations as well as environmental factors such as smoking and occupational exposure, are likely to contribute with an accumulative effect to risk.

There are several strengths of this study. The sample size is relatively large for a candidate gene study. The study population is homogeneous with minimal confounding of population structure. The patients were all histologically confirmed. The SNP panel is comprehensive. There are also a few limitations of this study. We used a false discovery rate (FDR) based approach to adjust for multiple testing and the FDR-adjusted P values were between 0.08 and 0.12 for the significant SNPs. A FDR threshold of 0.2 was suggested by previous studies for candidate gene studies [33]. Some of the associations are likely chance findings. Future external validations in independent studies are warranted to confirm the results of our studies. In addition, the CART analysis was exploratory and the results should be interpreted with caution. Nevertheless, our study strongly suggests that genetic variations in telomere maintenance genes modulate bladder cancer risk individually and jointly.

## Materials and Methods

### Ethics Statement

All patients signed a written informed consent and this study has been reviewed and approved by the Institutional Review Boards (IRB) of MD Anderson Cancer Center, Baylor College of Medicine, and Kelsey-Seybold Clinic.

### Study population and data collection

This study included bladder cancer patients who were recruited from The University of Texas MD Anderson Cancer Center and Baylor College of Medicine, recruitment starting in 1999. Cases were all histopathologically confirmed and previously untreated for chemotheraphy or radiotherapy pre-recruitment. There were no restrictions of recruitment on age, gender, or stage. Control subjects were recruited from Kelsey Seybold, the largest private multispecialty physician group in Houston. They were healthy individuals with no prior history of cancer except non-melanoma skin cancer, and were matched to patient cases by age (±5 years), sex, and ethnicity. Detailed questionnaire data including demographics, family history, smoking status, alcohol drinking, occupational history, and medical history were collected from all subjects through personal interview. Individuals who had smoked less than 100 cigarettes in their lifetimes were defined as never smokers, individuals who had smoked at least 100 cigarettes in their lifetime but had quit more than 12 months prior to diagnosis (cases) or interview (controls) were defined as former smokers, and individuals who were currently smoking or who had stopped <1 year prior were defined as current smokers. Former and current smokers were defined as ever smokers. Response rates for cases and controls were 92% and 76.7%, respectively. Because 90.6% of the patient population was Caucasian, we included only Caucasians in this study.

### SNP selection and genotyping

We selected 10 of the most important genes coding for proteins involved in telomere maintenance, including telomerase, shelterin proteins, and several telomere associated proteins, based on literature mining. Tagging SNPs were selected by the binning algorithm of LDSelect software (http://droog.gs.washington.edu/ldSelect.pl) (r^2^<0.8, MAF>0.05) within 10 kb upstream of the 5′ untranslated region (UTR) and 10 kb downstream of the 3′ UTR of each gene. We also included all the confirmed coding SNPs in the dbSNP database (http://www.ncbi.nlm.nih.gov/projects/SNP/). The final number of SNPs for each gene region was as follows: *PINX1*, 27; POT1, 8; PIP1, 1; *TEP1*, 42; TRF2, 2; TRF2IP, 2; TERT, 12; TNKS, 21; TNKS1BP1, 5; and TNKS2, 6. Genomic DNA was isolated from peripheral blood using the QIAamp DNA Blood Maxi Kit (Qiagen) according to the manufacturer's protocol. Genotyping was done using Illumina's iSelect custom SNP array platform according to the manufacturer's Infinium II assay protocol (Illumina). Genotyping data was then analyzed and exported using BeadStudio software (Illumina). The average call rate for the SNP array was >99%. Randomly selected 2% of samples were run in duplicates and the concordance of genotype calls was >99.9% for duplicated samples.

### Statistical analysis

Statistical analysis was performed using STATA 10.0 software (Stata Corp). χ^2^ test and Fisher's exact test were used to compare categorical variables, and Student's *t* test was used for continuous variables. Goodness-of-fit χ^2^ analysis was used to test Hardy-Weinberg equilibrium. Effects of SNP on bladder cancer risk was estimated as odds ratio (OR) and 95% confidence interval (CI). Unconditional multivariable logistic regression was performed under dominant, recessive, and additive models of inheritance adjusting for age, gender, and smoking status, where appropriate. False discovery rate (FDR) based Q value was calculated for individual SNP to adjust for multiple testing. We used a threshold of 0.20 for the Q value, previously suggested as more appropriate for moderate-sized studies with candidate gene approaches [33]. Haplotype analysis was conducted on SNPs of the *TEP1* gene.

For the cumulative effect of multiple SNPs on cancer risk, SNPs with significant association (P value for best fitting model <0.01) were evaluated. Using the subject group without any unfavorable genotypes as the reference, ORs and 95% CIs were calculated for the other groups using unconditional multivariate logistic regression adjusted for age, gender, smoking status and pack years. Unfavorable genotypes were sub-categorized into 3 groups (low-, medium-, and high-risk) according to number of unfavorable genotypes. The reference group was one without any unfavorable genotypes. High-order gene-gene interactions were explored via Classification and Regression Tree (CART) analysis, performed using HelixTree Genetics Analysis Software (v. 4.1.0, Golden Helix). Briefly, CART uses recursive partitioning to create a decision tree enabling identification of different combinations of variables at varying levels of risk. Analysis starts with the root node with all cases and controls, determines the most optimal split, i.e. smallest P value, for each following node, with multiplicity-adjusted P values to control tree growth (p<0.05). The process continues until terminal nodes have no statistically significant split or reach a predetermined minimum size. ORs and 95% CIs for each terminal node were calculated using logistic regression. P value≤0.05 was considered to be the threshold for significance in this study; all statistical analyses were two-sided.
